# Grief rumination mediates the association between self-compassion and psychopathology in relatives of missing persons

**DOI:** 10.1080/20008198.2017.1378052

**Published:** 2017-10-16

**Authors:** Lonneke I. M. Lenferink, Maarten C. Eisma, Jos de Keijser, Paul A. Boelen

**Affiliations:** ^a^ Department of Clinical Psychology and Experimental Psychopathology, Faculty of Behavioral and Social Sciences, University of Groningen, Groningen, The Netherlands; ^b^ Department of Clinical Psychology, Faculty of Social Sciences, Utrecht University, Utrecht, The Netherlands; ^c^ Arq Psychotrauma Expert Group, Diemen, The Netherlands

**Keywords:** Bereavement, loss, trauma, missing person, compassion, repetitive thinking, duelo, pérdida, trauma, persona fallecida, compasión, pensamiento repetitivo, 丧亲, 丧失, 创伤, 失踪人口, 同情, 重复思维, • This is the first study that examined the associations between self-compassion and emotional distress in the context of grief and loss., • Relatives of missing persons with more self-compassion experience less emotional distress., • The buffering effect of self-compassion on emotional distress may be explained by its dampening effect on ruminative thoughts related to the disappearance.

## Abstract

**Background**: The disappearance of a loved one is a unique type of loss, also termed ‘ambiguous loss’, which may heighten the risk for developing prolonged grief (PG), depression, and posttraumatic stress (PTS) symptoms. Little is known about protective and risk factors for psychopathology among relatives of missing persons. A potential protective factor is self-compassion, referring to openness toward and acceptance of one’s own pain, failures, and inadequacies. One could reason that self-compassion is associated with lower levels of emotional distress following ambiguous loss, because it might serve as a buffer for getting entangled in ruminative thinking about the causes and consequences of the disappearance (‘grief rumination’).

**Objective**: In a sample of relatives of missing persons we aimed to examine (1) the prediction that greater self-compassion is related to lower symptom-levels of PG, depression, and PTS and (2) to what extent these associations are mediated by grief rumination.

**Method**: Dutch and Belgian relatives of long-term missing persons (*N* = 137) completed self-report measures tapping self-compassion, grief rumination, PG, depression, and PTS. Mediation analyses were conducted.

**Results**: Self-compassion was significantly, negatively, and moderately associated with PG, depression, and PTS levels. Grief rumination significantly mediated the associations of higher levels of self-compassion with lower levels of PG (a*b = −0.11), depression (a*b = −0.07), and PTS (a*b = −0.11). Specifically, 50%, 32%, and 32% of the effect of self-compassion on PG, depression, and PTS levels, respectively, was accounted for by grief rumination.

**Conclusions**: Findings suggest that people with more self-compassion experience less severe psychopathology, in part because these people are less strongly inclined to engage in ruminative thinking related to the disappearance. Strengthening a self-compassionate attitude using, for instance, mindfulness-based interventions may therefore be a useful intervention to reduce emotional distress associated with the disappearance of a loved one.

## Abstract (es)

**Planteamiento:** La desaparición de un ser querido es un tipo único de pérdida, también llamada ‘pérdida ambigua’, la cual puede aumentar el riesgo de desarrollar duelo prolongado (DP), depresión y síntomas de estrés postraumático (EPT). Se sabe poco sobre los factores de protección y de riesgo de la psicopatología entre los familiares de las personas fallecidas. Un factor de protección potencial es la compasión hacia uno mismo, que hace referencia a estar abierto a, y aceptar, el dolor, los fracasos y las deficiencias personales. Se podría pensar que la compasión hacia uno mismo está asociada con niveles inferiores de angustia emocional después de una pérdida ambigua, ya que podría servir como amortiguador para no enredarse en el pensamiento repetitivo sobre las causas y consecuencias de la desaparición (denominado ‘ruminación del duelo’).

**Objetivo:** En una muestra de familiares de personas fallecidas buscamos examinar (1) la predicción de que una mayor compasión hacia uno mismo está relacionada con niveles de síntomas más bajos de DP, depresión y EPT, y (2) en qué medida estas asociaciones están mediadas por la ruminación del duelo.

**Método:** Los parientes holandeses y belgas de las personas fallecidas hace tiempo (N = 137) rellenaron auto-informes de medición relacionados con la compasión hacia uno mismo, la ruminación del duelo, el DP, la depresión y el EPT. Se realizaron análisis de mediación.

**Resultados:** La compasión hacia uno mismo se asoció de forma significativa, negativa y moderada con DP, depresión y niveles de EPT. La ruminación del duelo medió significativamente las asociaciones de niveles más altos de compasión hacia uno mismo con niveles más bajos de DP (a * b = −0,11), depresión (a * b = −0,07) y EPT (a * b = −0,11). Específicamente, el 50%, el 32% y el 32% del efecto de la compasión hacia uno mismo sobre el DP, la depresión y los niveles de EPT, respectivamente, se explicó por la ruminación del duelo.

**Conclusiones:** Los resultados sugieren que las personas con más compasión hacia uno mismo experimentan una psicopatología menos grave, en parte porque estas personas son menos inclinadas a participar en el pensamiento ruminativo relacionado con el fallecimiento. Fortalecer una actitud de compasión hacia uno mismo, por ejemplo, mediante intervenciones basadas en la conciencia plena, puede ser una intervención útil para reducir el sufrimiento emocional asociado con la desaparición de un ser querido.

## Abstract (zh)

**背景：**亲人的失踪是一种特殊的丧失，也被称作‘模糊的丧失’，可能会提高发生延长哀伤（PG）、抑郁和创伤后应激症状（PTS）的风险。对于失踪人口家属的精神病理学保护性和风险性因素我们知之甚少。一个潜在的保护性因素是自我同情（是指对自己痛苦、失败和无能的一种开放和接纳）。这可能是因为‘模糊的失去’之后的自我同情和更低水平情感痛苦有关，因为它可以作为一种缓冲使其免于陷入对亲人失踪的原因和结果的反刍思维（可称为‘哀伤反刍’）。

**目标：**在一个失踪人口样本中，我们想检验：（1）更高水平的自我同情和低水平延长哀伤、抑郁和PTS的关联；（2）哀伤反刍对这种关联性的中介作用能达到什么水平。

**方法：**荷兰与比利时长期失踪人口的亲属（N=137），完成自我同情、哀伤反刍、延长哀伤、抑郁和创伤后应激的自我报告量表，并进行中介效应分析。

**结果：**自我同情和PG显著关联、和抑郁负相关、和PTS水平有中度相关。哀伤反刍分别显著中介了对更高水平的自我同情和低水平的PG的关系（a*b = −0.11），和抑郁的关系（a*b=-0.07），和PTS的关系（a*b = −0.11）。尤其是，哀伤反刍可以分别解释50%、32%和32%的自我同情在PG、抑郁和PTS的作用。

**结论：**结果提示有更多自我同情的人经历更少的严重精神疾病，一部分是因为这些人更少被和失踪有关的反刍思维所困。使用例如以正念为基础的干预方法加强自我同情，可能因此成为一种有效的干预来减少亲人失踪产生的情绪痛苦。

The death of a significant other is a painful life event and may give rise to psychopathology, including posttraumatic stress (PTS; 11.8%; Onrust & Cuijpers, 2006), depression (21.9%; Onrust & Cuijpers, 2006), and symptoms of prolonged grief (PG; 9.8%; Lundorff, Holmgren, Zachariae, Farver-Vestergaard, & O’Connor, 2017). People confronted with a potential traumatic loss (e.g. homicide or suicide) are particularly susceptible to elevated post-loss psychopathology levels (Kristensen, Weisæth, & Heir, 2012). The long-term disappearance of a close relative is a unique type of potentially traumatizing loss (also coined an ‘ambiguous loss’; Boss, 2006). The psychological consequences of this loss for those left behind have barely been researched (see for an overview Lenferink, de Keijser, Wessel, de Vries, & Boelen, 2017).

There is some evidence that relatives of missing persons are at heightened risk to develop psychopathology. For instance, prevalence rates of clinically significant symptom levels of PG (23.3%), depression (68.5%), and PTS (67.1%) were high among people whose relative disappeared in the context of political repression on average 13 years earlier (Heeke, Stammel, & Knaevelsrud, 2015). Another study also showed high prevalence rates of PG (47.0%) and PTS (23.1%) among people whose relative disappeared due to various reasons (e.g. voluntarily missing or presumed homicide without a body) on average 16 years earlier (Lenferink, van Denderen, de Keijser, Wessel, & Boelen, 2017). To the best of our knowledge, no studies have yet examined variables associated with psychopathology among relatives of missing persons that could potentially be modified in treatment. Gaining insights in these variables is important for the development and refinement of treatment options.

A growing body of research suggests that self-compassion is positively associated with well-being (for an overview see Zessin, Dickhäuser, & Garbade, 2015) and negatively associated with depression (e.g. Costa & Pinto Gouveia, 2011; Gilbert, McEwan, Matos, & Rivis, 2011; Kuyken et al., 2010; Neff, Pisitsungkagarn, & Hseih, 2008; Raes, 2010, 2011; Roemer et al., 2009; van Dam, Sheppard, Forsyth, & Earleywine, 2011), anxiety (e.g. Costa & Pinto Gouveia, 2011; Gilbert et al., 2011; Neff, 2003; Raes, 2010; Roemer et al., 2009; van Dam et al., 2011), and (posttraumatic) stress (e.g. Costa & Pinto Gouveia, 2011; Dahm et al., 2015; Gilbert et al., 2011; Hiraoka et al., 2015; Raque-Bogdan, Ericson, Jackson, Martin, & Bryan, 2011; Thompson & Waltz, 2008). Self-compassion is defined as the tendency to be open to one’s own pain and suffering, to experience feelings of kindness toward oneself, to recognize that one’s experience is part of a common human experience, and to take an understanding, non-judgmental attitude toward one’s failures and inadequacies (Neff, 2003).

Being self-compassionate seems particularly helpful for people who have faced potentially traumatic events, such as the death of a significant other. Studies have shown that people exposed to traumatic events who showed higher levels of self-compassion reported lower levels of PTSD both concurrently (Dahm et al., 2015; Hiraoka et al., 2015; Thompson & Waltz, 2008) and longitudinally (Hiraoka et al., 2015; Zeller, Yuval, Nitzan-Assayag, & Bernstein, 2015). Furthermore, a laboratory study showed that a self-compassionate attitude toward a previous adverse life event can be induced and, once induced, leads to less emotional distress during the retrieval of memories of this event (Leary, Tate, Adams, Allen, & Hancock, 2007). Finally, clinical trials with PTSD patients have shown that self-compassion can be increased in treatment and promotes recovery from trauma (Beaumont, Galpin, & Jenkins, 2012; Hoffart, Øktedalen, & Langkaas, 2015; Kearney et al., 2013).

Strengthening of a self-compassionate attitude is an important aspect of mindfulness-based treatments (MacBeth & Gumley, 2012). Mindfulness and self-compassion are both rooted in Buddhist traditions and conceptually related. However, the targets of mindfulness and self-compassion differ. In general, mindfulness refers to present moment awareness to any inner experiences, whereas self-compassion is targeted at embracing one’s own suffering (Neff & Dahm, 2015). Preliminary findings of small clinical trials among bereaved people indicate that mindfulness-based approaches might be equally beneficial for targeting psychopathology levels in bereaved people as in non-bereaved people who suffer from similar complaints such as depression (O’Connor, Piet, & Hougaard, 2014; Thieleman, Cacciatore, & Hill, 2014). However, the link between self-compassion and psychopathology after bereavement, including PG symptoms, has, to the best of our knowledge, never been studied.

Although there is evidence that self-compassion is related to positive outcomes in people confronted with adverse life events, and it is conceivable that self-compassion has similar beneficial effects for bereaved people, less is known about mechanisms that may underlie the association between self-compassion and psychopathology (Raes, 2010). Exploring these mechanisms could further our understanding about the role of self-compassion in recovery from loss and trauma and could help to improve interventions fostering self-compassion in the treatment of trauma- and loss-related distress.

One possible explanation for the beneficial role of self-compassion in dealing with traumatic events is that self-compassion is associated with engagement with, rather than avoidance of, painful thoughts, memories, and feelings (Leary et al., 2007; Thompson & Waltz, 2008; Zeller et al., 2015). Accordingly, some researchers have argued that self-compassionate people ‘may be more likely to experience a natural process of exposure to trauma-related stimuli’ (Thompson & Waltz, 208, pp. 558). Exposure to loss-related stimuli is a critical ingredient of effective grief treatment (Bryant et al., 2014). Similarly, theoretical (Boelen, van den Hout, & van den Bout, 2006; Maccallum & Bryant, 2013; Stroebe & Schut, 1999) and empirical work (Boelen, de Keijser, & Smid, 2015; Boelen & van den Bout, 2010; Eisma et al., 2013; Schnider, Elhai, & Gray, 2007) emphasized that strategies to avoid and minimize engagement with painful feelings and thoughts associated with the loss are key to the onset and maintenance of psychopathology following the loss of a loved one.

One such avoidance strategy is repetitive negative thinking about the causes and consequences of the loss. This is called ‘grief rumination’ (Eisma, Schut et al., 2014). In contrast to Nolen-Hoeksema’s (2001) view, rumination may not be a maladaptive confrontational coping style, but could instead serve to refrain from admitting the loss and adjusting to it (Boelen, 2006; Stroebe et al., 2007). For example, ruminative thinking about why the loss occurred, how it could be prevented, and how best to respond to it could suppress more painful loss-related thoughts (e.g. about the true consequences of the loved one never coming back; Boelen, 2006; Eisma et al., 2013; Stroebe et al., 2007).

Grief rumination may concern different issues, including (1) counterfactuals about the loss (i.e. imagining alternative realities in which the loss could have been prevented), (2) reactions of others to the loss, (3) the unfairness of the loss, (4) the meaning and consequences of the loss, and (5) thoughts about one’s (emotional) reactions to the loss (Eisma, Stroebe et al., 2014). Research demonstrated that the first four types of grief rumination were concurrently and/or longitudinally related to elevated symptom levels of PG and/or depression levels. Interestingly, ruminative thinking about one’s emotional reactions to the loss was unrelated to PG and depression levels concurrently, but predicted less PG and depression levels over time (Eisma et al., 2015).

It has been repeatedly shown that rumination is concurrently and longitudinally linked to elevated PG, depression, and PTS levels following loss (Eisma & Stroebe, 2017; Eisma et al., 2012, 2013, 2015; Eisma, et al., 2014; Ito et al., 2003; Morina, 2011; Nolen-Hoeksema, Parker, & Larson, 1994). It has been proposed that rumination might also be an important cognitive strategy that causes and/or maintains psychopathological symptomatology in relatives of missing persons (Boss, 2006; Heeke et al., 2015; Lenferink, Wessel, de Keijser, & Boelen, 2016). To our knowledge, this notion has never been studied. According to the goal-discrepancy theory, ruminative thoughts reflect concerns and goals that have not yet been attained. Furthermore, those who have more extreme or unattainable goals may be more inclined to ruminate (Ehring & Watkins, 2008; Martin & Tesser, 1989). Because the disappearance of a loved one is inherently linked to uncertainties (e.g. not knowing whether the person suffered or is alive or dead) that are uncontrollable (Boss, 2006), disappearances may give rise to pervasive ruminative thinking.

It has been argued that elevated self-compassion might serve as a buffer for getting entangled in rumination, thereby preventing the exacerbation of emotional distress (Leary et al., 2007; Thompson & Waltz, 2008; Zeller et al., 2015). Accordingly, cross-sectional studies have shown that people with higher levels of self-compassion are less inclined to ruminate (Neff, 2003; Svendsen, Kvernenes, Wiker, & Dundas, 2016). Two studies supported the mediating effect of rumination in the relation between self-compassion and depression and anxiety (Krieger, Altenstein, Baettig, Doerig, & Holtforth, 2013; Raes, 2010).

The current study was concerned with self-compassion, grief rumination, and psychopathology among relatives of missing persons. First, we tested the prediction that higher levels of self-compassion were related to lower PG, depression, and PTS levels (Hypothesis 1). Because there is evidence that self-compassion is equally related to different types of symptoms (e.g. depression, anxiety, and stress; MacBeth & Gumley, 2012) we had no hypotheses with regard to the relative strength of the associations of self-compassion with PG, depression, and PTS levels. Second, we tested the prediction that the associations between self-compassion and post-loss psychopathology would be mediated by grief rumination (Hypothesis 2; see Figure 1). An additional aim of our study was to explore to what extent different subtypes of grief rumination mediate the association between self-compassion and psychopathology. Based on prior work (Eisma et al., 2015), we expected that rumination concerning the counterfactuals about the disappearance, reactions of others to the disappearance, the unfairness of the disappearance, and the meaning and consequences of the disappearance, partially mediated the association between self-compassion and post-loss psychopathology levels (Hypothesis 3; see Figure 2).

**Figure 1.**
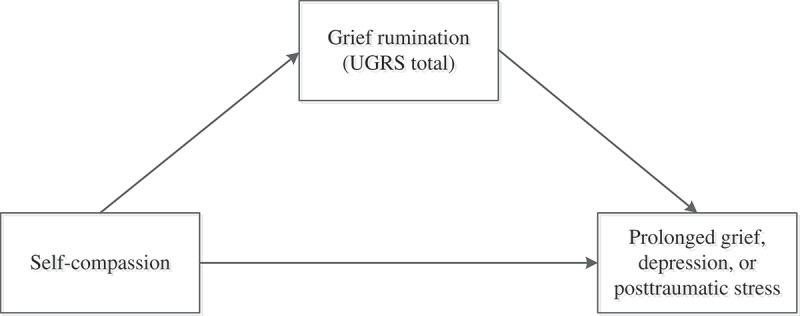
Single-mediation models. *Note*. We examined the potential mediating effect of grief rumination in the association between self-compassion and levels of prolonged grief (model 1), depression (model 2), and posttraumatic stress (model 3).

**Figure 2.**
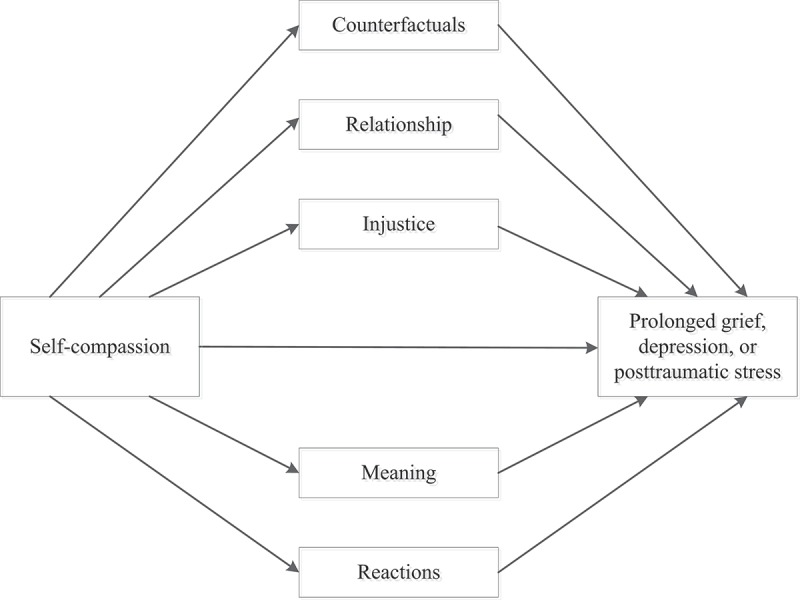
Multiple-mediation models. *Note*. We examined the potential mediating effect of subtypes of grief rumination in the association between self-compassion and levels of prolonged grief (model 1), depression (model 2), and posttraumatic stress (model 3).

## 1. Methods

### 1.1. Procedures

Data were used from an ongoing study about correlates and treatment of psychopathology in relatives of missing persons (Lenferink, van Denderen et al. 2017; Lenferink et al., 2016). Adults fluent in Dutch whose spouse, family member, or friend was missing for at least three months were eligible to participate. Data for the current study were collected between July 2014 and July 2016. The following definition of a missing person was used in the current study: ‘Anyone whose whereabouts is unknown whatever the circumstances of disappearance. They will be considered missing until located and their well-being or otherwise established’ (Association of Chief Police Officers, 2010, p. 15). Participants were recruited via invitation letters sent by different collaboration partners (i.e. [peer-] support organizations and the editorial office of a Dutch television show about missing persons), a website of the research project, and media coverage. In addition, people signing up for the study were asked to invite others. The ethical board of the University of Groningen approved this study. All participants gave written informed consent.

### 1.2. Measures

PG levels were assessed with the Inventory of Complicated Grief (ICG), which measures 19 grief reactions (Boelen, van den Bout, de Keijser, & Hoijtink, 2003; Prigerson et al., 1995). The ICG is together with the PG-13 a frequently used measure to assess grief reactions (Bui et al., 2015). We used the ICG because a validated Dutch translation of the PG-13 was not available at the time that the current study took place. Items were reformulated such that they referred to the disappearance (e.g. ‘I feel I have trouble accepting the disappearance’). Participants rated the presence of symptoms during the preceding month on a 5-point scale (0 = ‘never’ to 4 = ‘always’). Cronbach’s alpha in the current study was .92.

Depression severity was assessed with the 30-item Inventory of Depressive Symptomatology-Self-Report (IDS-SR; Rush, Gullion, Basco, Jarrett, & Trivedi, 1996). Each item represents a depressive symptom (e.g. ‘Falling asleep’), with four answer options ranging from 0 to 3 (e.g. answer option 0 = ‘I never take longer than 30 minutes to fall asleep’). Participants were instructed to select one option that best described how frequently they experienced the symptom during the past seven days. Cronbach’s alpha in the current study was .93.

PTS was assessed with the 20-item PTSD Checklist for DSM-5 (PCL-5; Blevins, Weathers, Davis, Witte, & Domino, 2015; Boeschoten, Bakker, Jongedijk, & Olff, 2014). The disappearance was the anchor-event; participants rated to what extent they experienced each symptom (e.g. ‘In the past month, how much were you bothered by feeling very upset when something reminded you of the events that are associated with the disappearance?’) on a 5-point scale ranging from 0 = ‘not at all’ to 4 = ‘extremely’. Cronbach’s alpha in the current study was .95.

Self-compassion was assessed with the 24-item Dutch Self-Compassion Scale (SCS; Neff, 2003; Neff & Vonk, 2009). Participants rated how often they behave in the stated manner on a 7-point scale ranging from 1 = ‘almost never’ to 7 = ‘almost always’ (e.g. ‘When I’m feeling down I tend to obsess and fixate on everything that’s wrong’ reverse scored). Cronbach’s alpha in the current study was .88.

Grief rumination was assessed with the Utrecht Grief Rumination Scale (UGRS; Eisma, Stroebe et al., 2014). Participants rated how often they experienced 15 ruminative thoughts (i.e. ‘How frequently in the past month did you … [ruminative thought]’) on a 5-point scale ranging from 1 = ‘never’ to 5 = ‘very often’. The total score was used in the current study as well as the scores on the five 3-item subscales. The subscale ‘Counterfactuals’ assesses counterfactual thinking about alternative past realities, related to the disappearance (e.g. ‘analyse whether you could have prevented his/her disappearance’; α = .78), the ‘Relationship’ subscale includes thoughts related to social responses to the disappearance (e.g. ‘query whether you receive the right support from family members’; α = .77), the ‘Injustice’ subscale includes thoughts about the unfairness of the disappearance (e.g. ‘think about the unfairness of this disappearance’; α = .73), the ‘Meaning’ subscale taps into thoughts about the consequences and meaning of the disappearance (e.g. ‘think how your life has been changed through his/her disappearance’; α = .83), and the ‘Reactions’ subscale assesses thoughts related to negative (emotional) reactions following the disappearance (e.g. ‘did you try to understand your feelings about the disappearance’; α = .76). Cronbach’s alpha for the total UGRS score was .91.

Higher scores on each measure were indicative of higher levels of psychopathology, self-compassion, and grief-related ruminative thinking. The wording that referred to ‘death’ or ‘stressful event’ in the above-mentioned measures were adapted to refer to the ‘disappearance’. The psychometric properties of all measures used in the current study are (at least) adequate (Blevins et al., 2015; Boelen et al., 2003; Eisma, Stroebe et al., 2014; Eisma et al., 2012; Neff, 2003; Rush et al., 1996).

### 1.3. Statistical analyses

Because the sum scores on the PG, depression, and PTS measures did not meet the assumption of normality (based on histograms and skewness and kurtosis values), non-parametric (Spearman’s rho) correlations were calculated between all variables. Mediation analyses were performed using the PROCESS plug-in for SPSS (Hayes, 2013). First, three single-mediation models were estimated with self-compassion as independent variable and PG, depression, or PTS levels as dependent variables, and the UGRS total score (denoting grief rumination) as mediator. Second, three multiple mediation models were tested using the same independent and dependent variables, and total scores of the five UGRS scales as mediator variables. In a prior study using the same data, time since disappearance (in years) and dichotomized kinship to the missing person (0 = missing person is child or spouse, 1 = other) were found to be significantly associated with levels of PG, depression, and PTSD (Lenferink, Wessel, & Boelen, submitted). Therefore, these two variables were included as covariates in the mediation models.

Unstandardized regression coefficients were estimated for each path of the mediation model. Path *a* reflects the effect of the independent variable (i.e. self-compassion) on the mediator (i.e. grief rumination total or subscale scores) while controlling for the covariates, path *b* represents the effect of the mediator on the dependent variable (i.e. PG, depression, or PTS levels) while controlling for the independent variable (and covariates), path *c* is the total effect of the independent variable on the dependent variable, and path *c’* is the direct effect of the independent variable on the dependent variable while controlling for the effect of the mediator(s) and covariates. Bias-corrected 95% bootstrap confidence intervals (BC 95% CI) for the indirect effect (a*b) of the independent variable on the dependent variable through the mediator(s) were computed based on 5000 bootstrap resamples. These indirect effects were considered statistically significant when zero was not included in the BC 95% CI. The proportion of the effect of the independent variable on the dependent variable explained by the mediator(s) was calculated by using the following formula: 1 – *c’*/*c* (MacKinnon, Fairchild, & Fritz, 2007). Less than 5% of the data was missing per item. Missing data were therefore imputed with the mean.

## 2. Results

### 2.1. Preliminary analyses

Socio-demographic and loss-related characteristics of the 137 participants are displayed in Table 1.

**Table 1.** Characteristics of the participants (*N* = 137).

Gender, *n* (*%*)	
Men	45 (32.8)
Women	92 (67.2)
Age (years), *M* (*SD*)	57.9 (14.1)
Educational level, *n* (*%*)	
Low	32 (23.4)
Middle	45 (32.9)
High	60 (43.8)
Lost relative is, *n* (*%*)	
Partner/spouse	18 (13.1)
Child	44 (30.7)
Parent	14 (10.2)
Sibling	31 (22.6)
Other family member	29 (21.2)
Other	3 (2.2)
Number of years since loss, *M* (*SD*)	15.2 (16.9)
Type of disappearance, *n* (*%*)	
Criminal act	44 (32.1)
Voluntarily	33 (24.1)
Accident	33 (24.0)
No specific suspicion	27 (19.7)
Unique victims	90 (65.7)
Recruitment via	
Editorial office of TV show about missing persons	36 (26.3)
Peer support organizations	31 (22.6)
Non-governmental support organization	21 (15.3)
Family or friends	37 (27.0)
Other	12 (8.8)

Correlations between variables are presented in Table 2. Self-compassion was inversely related to scores on the measures of psychopathology and grief rumination. Grief rumination scores were all positively related to the indices of psychopathology.

**Table 2.** Zero-order correlations between all variables (*N* = 137).

	2	3	4	5	6	7	8	9	10
1. Prolonged grief	.70***	.77***	−.35***	.79***	.62***	.63***	.69***	.68***	.60***
2. Depression		.83***	−.41***	.64***	.51***	.57***	.41***	.60***	.52***
3. Posttraumatic stress			−.46***	.70***	.55***	.57***	.54***	.63***	.58***
4. Self-compassion				−.29**	−.21*	−.25**	−.24**	−.32***	−.18*
5. UGRS total					.85***	.76***	.80***	.81***	.84***
6. UGRS Counterfactuals						.49***	.68***	.56***	.67***
7. UGRS Relationship							.44***	.60***	.60***
8. UGRS Injustice								.56***	.56***
9. UGRS Meaning									.62***
10. UGRS Reactions									

UGRS = Utrecht Grief Rumination Scale; * *p *< .05; ** *p *< .01; *** *p *< .001.

### 2.2. Single-mediation analyses

Table 3 shows the results of the mediation analyses with relevant covariates (i.e. kinship to the missing person and time since disappearance) being taken into account. It was found that higher levels of self-compassion were significantly associated with lower tendencies to ruminate about the disappearance (*a* = −0.14) and lower symptom levels of PG (*c* = −0.22), depression (*c* = −0.25), and PTS (*c* = −0.34). Furthermore, higher tendencies to ruminate were significantly related to increased symptom levels of PG (*b* = 0.79), depression (*b* = 0.51), and PTS (*b* = 0.75) when taking self-compassion into account. Zero was not included in the BC 95% CI’s of the indirect effects indicating that grief rumination significantly mediated the associations of higher levels of self-compassion with lower levels of PG (a*b = −0.11), depression (a*b = −0.07), and PTS (a*b = −0.11). In total, 50%, 32%, and 32% of the effect of self-compassion on PG, depression, and PTS levels, respectively, was accounted for by grief rumination.

**Table 3.** Mediation analyses (*n* = 136^1^).

Model	Mediator	*a*	*b*	Total effect *c*	Direct effect *c’*	Unique indirect effect a*b (BC 95% CI)	MacKinnon effect size
**Prolonged grief**							
Single mediation	UGRS total	−0.14**	0.79***	−0.22***	−0.11**	−0.11* (−0.20, −0.03)	.50
Multiple mediation	UGRS counterfactuals	−0.02	0.16		−0.09**	<-0.01 (−0.03, 0.01)	.59
	UGRS relationship	−0.03*	1.18***			−0.03* (−0.07, −0.01)	
	UGRS injustice	−0.03*	1.73***			−0.05* (−0.11, −0.01)	
	UGRS meaning	−0.04***	0.90**			−0.04* (−0.08, −0.01)	
	UGRS reactions	−0.02	0.15			<-0.01 (−0.03, 0.01)	
**Depression**							
Single mediation	UGRS total	−0.14**	0.51***	−0.25***	−0.17***	−0.07* (−0.13, −0.03)	.32
Multiple mediation	UGRS counterfactuals	−0.02	0.39		−0.16***	−0.01 (−0.05, 0.01)	.36
	UGRS relationship	−0.03*	0.85*			−0.02* (−0.07, <-0.01)	
	UGRS injustice	−0.03*	0.05			<-0.01 (−0.04, 0.03)	
	UGRS meaning	−0.04***	1.02*			−0.04* (−0.10, −0.01)	
	UGRS reactions	−0.02	0.30			−0.01 (−0.04, 0.01)	
**Posttraumatic stress**							
Single mediation	UGRS total	−0.14**	0.75***	−0.34***	−0.23***	−0.11* (−0.19, −0.03)	.32
Multiple mediation	UGRS counterfactuals	−0.02	0.34		−0.22***	−0.01 (−0.05, 0.01)	.35
	UGRS relationship	−0.03*	1.04*			−0.03* (−0.09, <-0.01)	
	UGRS injustice	−0.03*	1.02*			−0.03* (−0.10, <-0.01)	
	UGRS meaning	−0.04***	0.94			−0.04* (−0.10, <-0.01)	
	UGRS reactions	−0.02	0.51			−0.01 (−0.05, 0.01)	

Note. ^1^ = one participant did not fill in the date of the disappearance of his/her loved one and was therefore excluded from the mediation analyses; UGRS = Utrecht Grief Rumination Scale; *a* = the effect of X on M while controlling for the covariates; *b* = the effect of the mediator on Y, while controlling for X, other mediators, and covariates; *c* = the effect of X on Y; *c’* is the direct of X on Y while controlling for the mediator(s) and covariates; BC 95% CI = Bias corrected bootstrap confidence intervals (5000 resamples); * *p *< .05; ** *p *< .01; *** *p *< .001.

### 2.3. Multiple-mediation analyses

Table 3 also shows the results of the multiple-mediation models. The three UGRS subscales ‘Relationship’, ‘Injustice’, and ‘Meaning’ emerged as unique mediators of the association between self-compassion and symptom levels of PG and PTS. For depression, two mediators were significant, namely UGRS ‘Relationship’ and ‘Meaning’. All mediators combined accounted for 59%, 36%, and 35% of the effects of self-compassion on symptom levels of PG, depression, and PTS, respectively.

## 3. Discussion

The aim of the current study was to investigate the associations between self-compassion and psychopathology levels among people confronted with the long-term disappearance of a loved one. Moreover, we examined whether these associations were mediated by grief rumination.

In support of our first hypothesis, self-compassion was significantly and negatively associated with PG, depression, and PTS levels, even when taking grief rumination and relevant background variables into account. We found moderate correlations (*r*_s_ = −.35 to −.46) between self-compassion and psychopathology levels; correlations appear to be lower than the overall large correlation between psychopathology and self-compassion (*r* = −.54) found in a meta-analysis (MacBeth & Gumley, 2012). These previous large effects were predominantly based on non-clinical (student) samples. Correlations between self-compassion and PTS ranged from small to large in trauma exposed samples (Dahm et al., 2015; Hiraoka et al., 2015; Thompson & Waltz, 2008).

In line with our second hypothesis, the single-mediation models showed that the associations between self-compassion and PG, depression, and PTS levels were mediated by grief rumination. In other words, people who have stronger tendencies to approach their emotional pain in an open and understanding way (i.e. more self-compassion) are less likely to get entangled in ruminative thoughts related to the disappearance which, in turn, attenuates psychopathology levels. This accords with and extends previous research indicating that rumination mediates the linkage between self-compassion and depression and anxiety (Krieger et al., 2013; Raes, 2010). Although we did not test it directly, these findings also seem to support previous research denoting that self-compassion may be viewed as a way of exposure to internal threats (Krieger et al., 2013; Thompson & Waltz, 2008) and rumination as a way of avoidance of painful aspects of the loss (Eisma, Schut et al., 2014; Stroebe et al., 2007).

Partly in accordance with our third hypothesis, the multiple-mediation analyses indicated that ruminative thoughts about the meaning of the disappearance (i.e. UGRS meaning) and reactions of others to the disappearance (i.e. UGRS relationship) were significant mediators of the associations between self-compassion and PG, depression, and PTS levels. Thoughts about the unfairness of the disappearance (i.e. UGRS injustice) only significantly mediated the associations between self-compassion and PG and PTS levels. Contrary to our expectations, ruminative thinking about alternative past realities in which the person did not disappear (i.e. UGRS counterfactuals) was not a significant mediator. Although zero order correlations between counterfactual thinking and self-compassion and psychopathology were statistically significant, these associations disappeared once other variables (e.g. other rumination subtypes, time since disappearance) were taken into account. Previous studies also have shown that some types of rumination uniquely mediate the effect of self-compassion on depression and/or anxiety while others do not. For instance, Raes (2010) found that brooding but not reflection mediated the association between self-compassion and depression; Krieger et al. (2013) found that symptom-focused rumination but not self-focused rumination mediated the linkage of self-compassion with depression. Taken together, our findings suggest once more that some forms of ruminative thinking are more maladaptive than others when it comes to dealing with the loss of a loved one (Eisma et al., 2015; Stroebe et al., 2007).

It is important to note that grief rumination was a partial mediator in the current study. This indicates that other phenomena may also explain the associations between self-compassion and post-loss psychopathology levels including, for instance, adaptive emotion-regulation skills (e.g. the ability to tolerate unpleasant emotion; Diedrich, Burger, Kirchner, & Berking, 2016). Of course, many other factors may potentially be involved in the association between self-compassion and post-loss psychopathology (see for an overview Barnard & Curry, 2011). Following previous studies (see Krieger et al., 2013; Raes, 2010) we examined the mediating role of rumination in the association between self-compassion and psychopathology levels. However, we cannot preclude the possibility that self-compassion mediates the association between rumination and psychopathology. Future studies, preferably using a longitudinal design and clinical samples, may further study the temporal relationships between these constructs, for example by using cross-lagged analyses (see Krieger, Berger, & Holtforth, 2016).

A number of limitations need to be taken into account while interpreting our findings. First, the cross-sectional design precludes drawing conclusions about temporal precedence and causality. Second, due to composition of our convenience sample (i.e. a Dutch and Belgian community sample of relatives of long-term missing persons), the generalizability of our findings to relatives of missing persons in general, but also people confronted with other loss-experiences, with clinical levels of psychopathology, who are more recently bereaved, and have other cultural backgrounds may be limited. Third, self-report measures instead of diagnostic interviews were used, which may have led to overestimation of psychopathology levels (Engelhard et al., 2007). In addition, we did not take the nested structure of our data (i.e. the 137 participants were related to 90 unique missing persons) into account in the analyses. Because our sample included only a relatively small number of relatives of the same missing person, it is unlikely that this has increased the chance of Type I error. Lastly, our sample size was relatively small (see Fritz & MacKinnon, 2007), which may have increased the risk of Type II error.

Despite these limitations, the current study is the first that examined the associations between self-compassion and psychopathology levels in the context of grief and loss. Although more research is needed to draw firm conclusions about these associations among people confronted with the loss of a loved one, the results of our study suggest that fostering self-compassion in treatment might reduce post-loss psychopathology levels by reducing ruminative tendencies. There is evidence that enhancement of self-compassion and reduction of ruminative tendencies are mechanisms of change in mindfulness-based treatments for recurrent depression (van der Velden et al., 2015). Moreover, other third-wave cognitive behavioural treatments, such as compassion-focused therapy and acceptance and commitment therapy, are increasingly being used to target these phenomena (Beaumont & Hollins Martin, 2015; Dindo, Van Liew, & Arch, 2017). The first results of small trials among bereaved persons showed that mindfulness-based interventions might be effective in reducing symptoms of depression (O’Connor et al., 2014; Thieleman et al., 2014), anxiety, and PTS (Thieleman et al., 2014). Although non-significant reductions were found in PG and PTS levels in O’Connor et al.’s (2014) study and some participants in Thieleman et al.’s study (2014) reported increased symptomatology following the mindfulness-based treatment, it may be valuable to further study the potential effectiveness of these interventions, because the current treatment-of-choice, cognitive behavioural therapy, results in clinically relevant change in PG levels in at most 50% of people with PG (Doering & Eisma, 2016). Consequently, based on the current findings, we recommend further exploration of the value of self-compassion in recovery from loss.
